# The limits of human predictions of recidivism

**DOI:** 10.1126/sciadv.aaz0652

**Published:** 2020-02-14

**Authors:** Zhiyuan “Jerry” Lin, Jongbin Jung, Sharad Goel, Jennifer Skeem

**Affiliations:** 1 Stanford University, Stanford, CA, USA.; 2 University of California, Berkeley, Berkeley, CA, USA.

## Abstract

Dressel and Farid recently found that laypeople were as accurate as statistical algorithms in predicting whether a defendant would reoffend, casting doubt on the value of risk assessment tools in the criminal justice system. We report the results of a replication and extension of Dressel and Farid’s experiment. Under conditions similar to the original study, we found nearly identical results, with humans and algorithms performing comparably. However, algorithms beat humans in the three other datasets we examined. The performance gap between humans and algorithms was particularly pronounced when, in a departure from the original study, participants were not provided with immediate feedback on the accuracy of their responses. Algorithms also outperformed humans when the information provided for predictions included an enriched (versus restricted) set of risk factors. These results suggest that algorithms can outperform human predictions of recidivism in ecologically valid settings.

## Teaser

Statistical algorithms can outperform human predictions of recidivism.

## INTRODUCTION

Algorithms and predictive analytics inform decisions in almost every sector of public policy, including criminal justice. When judges, correctional authorities, and parole boards make decisions regarding incarceration, supervision, and release, they now routinely turn to risk assessment instruments (RAIs), which are checklists that summarize “risk factors” for estimating a person’s likelihood of future reoffending. The chief rationale is a belief that RAIs outperform unaided human judgment in predicting recidivism (*1*, *2*).

The validity of this rationale, however, has been questioned. In a recent high-profile study, Dressel and Farid (*3*) found that a widely used RAI called Correctional Offender Management Profiling for Alternative Sanctions (COMPAS) “is no more accurate … than predictions made by people with little or no criminal justice expertise.” The authors recruited 400 online participants through Amazon’s Mechanical Turk platform to take part in their study. They showed each participant 50 short descriptions of real defendants drawn from a publicly available COMPAS dataset and asked participants to indicate whether they thought each defendant would commit another crime within 2 years. Averaging across these responses, the overall accuracy of participants was 62%, comparable to the accuracy of algorithmic COMPAS predictions (65%). [Aside from effectiveness, some have also questioned the equity of RAIs. See, for example, Angwin *et al.* (*4*) and responses to those critiques (*5*–*9*).]

However, a closer look at the Dressel and Farid study suggests that laypeople’s predictions were elicited in a manner that may not best represent unaided human judgment, particularly the kind that judges, probation officers, and other professionals must exercise when predicting reoffending in the real world. More specifically, the study design focused people’s attention on the most predictive factors and promoted learning over the course of the experiment, perhaps boosting accuracy rates as a result. In a new series of experiments, we tested the impact of three conditions on the relative accuracy of human judgment and RAIs in predicting reoffense. Collectively, these experiments were designed to illuminate both the situations in which humans can predict recidivism as accurately as algorithms and settings in which algorithms can provide better estimates than humans.

First, we tested the impact of providing “streamlined” versus “enriched” information for prediction. Dressel and Farid provided people with brief vignettes that listed five risk factors for recidivism per case in narrative form: the individual’s sex, age, current charge, and number of prior adult and juvenile offenses. This format mimics structured checklists of selective risk factors that have been shown to increase professionals’ ability to make accurate predictions (*10*). However, the information available in justice settings is far less constrained. Presentence investigation reports, attorney and victim impact statements, and an individual’s demeanor all add complex, inconsistent, risk-irrelevant, and potentially biasing information. We hypothesized that statistical tools predict better than humans when both are provided with more complex or otherwise noisy risk information.

We tested this hypothesis by manipulating whether streamlined information (Dressel and Farid’s 5 risk factors) or enriched information (those 5 factors plus 10 more) was provided. We ensured that all information was consistent and risk relevant. Given that the COMPAS dataset lacked these additional risk factors, we used similar datasets on an RAI called the “level of service inventory–revised” (LSI-R) (*11*). The LSI-R includes, for example, information on one’s criminal history, employment status, and substance use. For each of the 10 risk factors assessed by the LSI-R, we wrote phrases to describe each score on that factor (e.g., “has a serious drinking problem that interferes with work” for a substance abuse score of 3). These phrases were combined to create enriched vignettes that described many aspects of real individuals, as a contrast to streamlined vignettes that only described the most predictive risk factors for the same individuals.

Second, we tested the impact of providing people with feedback on their accuracy across a series of trials. In each of the 50 rounds of Dressel and Farid’s study, participants made a prediction, were informed whether the prediction was correct (and their cumulative accuracy), and then moved on to the next vignette. In other words, prediction events were experienced sequentially, with immediate feedback on accuracy. This created a “kind” environment, one shown to be ideal for humans to intuitively learn the probabilities of specific outcomes, even when the rules are not transparent (*12*). Kind environments can promote accuracy, unlike the “wicked” learning environments that characterize most justice settings, where outcomes cannot be observed immediately or are never observed at all (*10*, *13*). In the absence of such feedback, we hypothesized that algorithms predict better than humans. We tested this hypothesis by manipulating whether people were provided with feedback on their accuracy using both the COMPAS dataset and our LSI-R datasets.

Third, we tested the impact of base rates, a group’s overall probability of reoffending, on the relative predictive accuracy of algorithms and humans. Base rates vary substantially across contexts. For example, in the COMPAS data used by Dressel and Farid, the base rate of rearrest for any type of crime is 48%, whereas the base rate of rearrest for violent crime in the same dataset is only 11%. Even when people are explicitly told base rates, they often fail to update their prior beliefs, a phenomenon called “base rate neglect” (*14*–*17*). Statistical algorithms, in contrast, are designed to incorporate this information accurately and consistently. For this reason, we expect the accuracy of human predictions, but not the accuracy of algorithmic decisions, to be particularly sensitive to base rates.

There is one common exception to this expectation. People often do take base rates into account when the probabilities are “directly experienced through trial-by-trial outcome feedback” (*18*). Dressel and Farid’s feedback protocol creates this kind of intuitive learning environment. When feedback is provided, we thus expect the accuracy of humans to be substantially less sensitive to base rates. We tested these hypotheses by varying the base rate of recidivism in both the COMPAS dataset (using any versus violent rearrest) and the LSI-R datasets (where base rates differ by location), across feedback conditions.

## Design

**Fig. 1:**
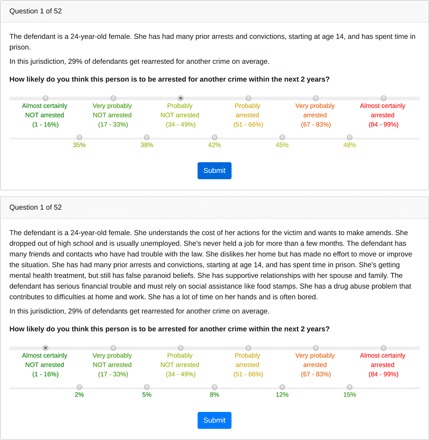
Sample vignettes. The top and bottom panels provide examples of streamlined and enriched vignettes, respectively. Participants assessed the likelihood of re-arrest on a 30-point scale, as shown in each panel.

Following Dressel and Farid, we recruited participants on Amazon’s Mechanical Turk platform to estimate the likelihood that defendants would be rearrested within 2 years of release on the basis of brief descriptions of the individuals. In the original study, participants were simply asked for binary yes/no predictions of recidivism. We altered this design to instead elicit predictions on a 30-point probability scale. To do so, as shown in Fig. 1, we first asked participants to select one of six risk buckets, ranging from “almost certainly NOT arrested (1 to 16%)” to “almost certainly arrested (84 to 99%).” On the basis of this initial response, we then asked individuals to select one of five subcategories to obtain more specific probability estimates. For example, in the lowest risk bucket, the subcategories were 2, 5, 8, 12, and 15%. Participants could not indicate exactly 50% likelihood of rearrest, so reported probabilities could unambiguously be converted to binary predictions based on a 50% probability threshold.

We extended the original study in three additional ways. First, whereas Dressel and Farid focused on a single dataset, we repeated our experiments on four: (i) COMPAS balanced base rate assessments of any recidivism in Broward County, FL (the dataset used by Dressel and Farid); (ii) COMPAS low base rate assessments of violent recidivism, also in Broward County; (iii) LSI-R balanced base rate assessments of recidivism in a midwestern state; and (iv) LSI-R low base rate assessments of recidivism in a southwestern state. In the first three datasets, “recidivism” means rearrest; in the fourth, recidivism means reincarceration. A summary of these four datasets is presented in Table 1. Second, we examined the effects of immediate feedback on human predictions. In the original study, participants were told after each prediction whether a defendant was indeed rearrested. We instead randomly assigned participants either to receive or not to receive feedback. Last, we investigated the effects of information richness on predictive accuracy. In the two COMPAS datasets, including the dataset used in the original study, relatively little information is available about individuals, and that which is available (e.g., age, gender, and number of past arrests) is strongly associated with recidivism risk. Vignettes based on COMPAS datasets are necessarily streamlined (i.e., restricted to the five risk factors available). However, in the two LSI-R datasets, we have more complete information on each individual, including 10 additional risk factors related, for example, to education, employment, and substance use. Vignettes based on LSI-R datasets can be streamlined like those presented in the original study or enriched with descriptions containing more detailed information.

**Table 1:** Characteristics of the four datasets that we considered. BR, base rate.

	**COMPAS balanced BR**	**COMPAS low BR**	**LSI-R balanced BR**	**LSI-R low BR**
Number of cases	1000	1000	311	1954
BR of recidivism	48%	11%	29%	9%
Features	Streamlined	Streamlined	Streamlined/enriched	Streamlined/enriched
Number of responses (no feedback)	2700	2400	2850/2500	2850/2900
Number of responses (feedback)	2400	3000	2700/2400	3000/2550

In summary, we carried out four separate experiments, one for each of the four datasets that we considered. In the two COMPAS experiments, participants were randomly assigned to receive or not to receive feedback. In the two LSI-R experiments, participants were randomly assigned to one of the two feedback conditions and independently assigned to see streamlined or enriched vignettes, in a 2 by 2 design. In all cases, participants provided 50 predictions and received financial compensation for accuracy, in line with the original study. In aggregate, across all the experiments, we collected 32,250 responses from 645 participants.

## RESULTS

As detailed below, we compared human predictions of recidivism to those from existing tools (COMPAS and LSI-R); we also fit our own statistical models to the data as another point of comparison. Predictions from existing tools and our own models were restricted to providing responses on the same 30-point probability scale available to study participants. Similar to Dressel and Farid, we quantified performance both in terms of binary classification accuracy, which we henceforth simply call “classification accuracy,” and ranking accuracy, as measured, in part, by the area under the receiver operating characteristic curve [commonly called “area under the curve” (AUC)].

### Classification accuracy

**Fig. 2:**
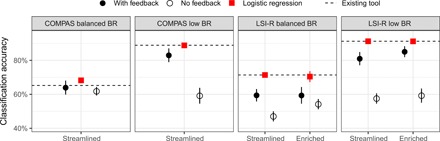
Classification accuracy of human predictions, statistical models, and existing tools. Classification accuracy is shown for (i) human predictions, with and without immediate feedback; (ii) a logistic regression model that we trained using the same information provided to study participants; and (iii) the existing tools, COMPAS or LSI-R. For participants in the feedback condition, only the last 10 responses for each participant were used, to account for the effects of learning. Error bars represent 95% confidence intervals and are typically smaller than the height of red squares for the logistic regression models.

Figure 2 shows the classification accuracy of study participants (with and without feedback), existing tools, and our own statistical models. In particular, the solid circle in the leftmost panel corresponds to the setting considered in Dressel and Farid: the COMPAS dataset, with immediate feedback provided to participants. In line with that study, we found that participants performed on par with the COMPAS algorithm and with our own logistic regression model: 64% accuracy for participants; 65% for COMPAS, indicated by the dashed line; and 68% for our model, indicated by the red square. Furthermore, even without immediate feedback (open circle, 62%), the study participants did reasonably well. We were thus largely able to replicate the main result reported by Dressel and Farid despite some differences in experiment design (e.g., we elicited probability judgments rather than binary predictions). See also tables S1 to S3 for further statistical comparisons between the accuracy of humans and algorithms.

However, we saw qualitatively different patterns in the three other datasets that we considered. In those datasets, as shown in Fig. 2, study participants performed consistently worse than both the existing RAIs (COMPAS and LSI-R) and our own logistic regression models, with a particularly large performance gap when feedback was not provided. For example, in the COMPAS low base rate dataset, COMPAS and our logistic regression model both achieved 89% classification accuracy, but participants attained only 83% accuracy when provided with feedback, and participants attained only 60% accuracy without feedback. Figure S3 shows that classification accuracy improved over time when feedback was provided, but performance did not improve fast enough for participants to be competitive with the existing tools or our statistical models. (To adjust for these learning gains, in the feedback condition, we report accuracy on the final 10 of the 50 questions answered by a participant.)

The effect of feedback on participants’ performance appears most pronounced in the datasets with relatively low base rates of recidivism. In the COMPAS balanced base rate data used by Dressel and Farid, 48% of defendants recidivated. In comparison, base rates are 11, 9, and 29% in the COMPAS low base rate, LSI-R low base rate, and LSI-R balanced base rate datasets, respectively. In these latter three datasets, participants consistently overestimated risk, hurting their classification accuracy (see fig. S4 and table S3), despite the fact that study participants were explicitly and repeatedly informed of the lower base rates, as shown in Fig. 1.

Last, and in contrast to providing feedback, we found that providing enriched information for predictions had minimal effect on classification accuracy. As shown in Fig. 2 for the two LSI-R datasets, both human participants and our own statistical models showed little to no improvement in classification accuracy when given more information on which to base judgments, in the enriched condition (see also table S5).

### Ranking accuracy

Classification accuracy is a useful starting point, but it can also be a problematic measure of performance in unbalanced datasets. For example, in the COMPAS low base rate dataset (with 11% recidivism), COMPAS and our own statistical model have about the same accuracy as a naive classifier that predicts that no one recidivates, although human participants performed considerably worse than even this simple classifier. We thus next gauge performance in terms of AUC, a popular measure that mitigates this issue of class imbalance. Loosely, AUC measures the extent to which predictions correctly rank individuals by risk, ignoring the absolute stated risk level. To more formally define AUC, suppose, in a given dataset, that *X*_1_ is a randomly selected individual who ultimately recidivates and *X*_0_ is a randomly selected individual who ultimately does not. Then, AUC is Pr (*r*(*X*_1_) ≥ *r*(*X*_0_)), where *r*(*x*) is the reported probability that *x* recidivates.

**Fig. 3:**
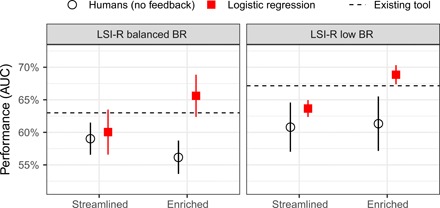
Ranking accuracy of human predictions, statistical models, and existing tools. Ranking accuracy, as measured by AUC, is shown for (i) human predictions without feedback, (ii) logistic regression models that use the same information provided to study participants, and (iii) the existing LSI-R tools. Error bars indicate 95% confidence intervals.

Figure 3 shows the ranking accuracy of existing tools, our own statistical models, and human predictions without feedback, as measured by AUC for the two LSI-R datasets, where the enriched condition is available. We restrict our attention to the no-feedback condition because humans with feedback update their estimates of base rates over time (fig. S3), making it difficult to accurately assess risk rankings since the scale of predictions changes across responses. We found that study participants had worse ranking accuracy than the LSI-R RAI. (We saw similar results for the two COMPAS datasets, as shown in fig. S1.) Furthermore, as was the case for classification accuracy, in Fig. 2, humans showed little to no improvement in ranking accuracy when provided with enriched information (see also table S2).

Our logistic regression model, however, did improve in ranking accuracy when provided with more information, a pattern that was not apparent when performance was measured via classification accuracy, as shown in Fig. 2. In the LSI-R datasets, very few individuals have estimated likelihood of recidivism that exceeds 50%, so the optimal binary prediction is “no recidivism” in almost every case, even when provided with the enriched information. For this reason, classification accuracy is too coarse a measure to reveal the value of additional information. Ranking accuracy, in contrast, allows for more nuanced distinctions among predictions, which, in turn, reveals the gap between risk assessments based on streamlined and enriched information (see table S5).

Our findings further suggest that humans perform relatively well when a very limited amount of information is sufficient to create accurate risk rankings. For example, when age and number of past offenses is all one needs to assess risk, it is reasonable that appropriately motivated humans can compete with statistical models. In the cases that we consider, human participants generally performed on par with our logistic regression models that were based on limited information, in the streamlined condition, but when more information was useful, the models appropriately incorporated that information while the human participants often did not, as seen in the enriched condition. In both panels of Fig. 3, the gap between humans and the logistic regression models is wider in the enriched condition (see also table S4).

**Fig. 4:**
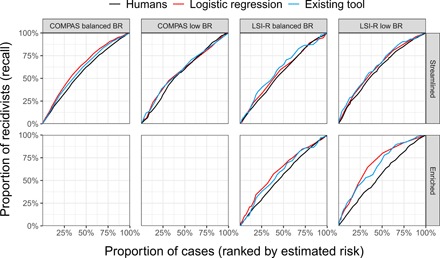
An alternative measure of ranking accuracy. Proportion of people who recidivated that were identified when ranking by the risk assessments of humans in the no-feedback condition, a logistic regression model, and existing tools (COMPAS or LSI-R). For each value p on the horizontal axis, the vertical axis shows the proportion of all recidivists that are included among the *p*-percent of the population deemed riskiest. Human performance was generally comparable to algorithmic tools in the streamlined condition (**top**), but algorithmic tools outperformed humans when more information was made available [enriched condition, (**bottom**)].

Last, we took a complementary, cost-benefit approach to assess ranking performance. Suppose that a policy-maker aims to allocate limited resources (e.g., community supervision) to those individuals deemed most likely to recidivate. To assess the performance of different ranking strategies, one can compute the proportion of recidivists that are listed in the top *p*-percent of candidates in each strategy. (This measure is also known as “recall at *p*” in the machine learning literature.) Figure 4 traces out the corresponding curves for humans and algorithms in our experiments, for all values of *p* from 0 to 100% on the horizontal axis. As with our AUC analysis, we restricted to study participants who did not receive feedback. In line with those results above, we found that study participants in the streamlined condition generally performed on par with existing tools and with our own statistical models but that algorithms outperformed humans when more information was available. For example, in the LSI low base rate dataset in the enriched condition, the top 50% of individuals deemed riskiest by study participants in reality contained 58% of recidivists in the dataset, just slightly better than random. In comparison, the top 50% deemed riskiest by LSI-R and our own statistical model contained 74 and 80% of recidivists, respectively. Similarly, in the LSI balanced base rate dataset in the enriched condition, the top 50% deemed riskiest by study participants contained 57% of recidivists, while LSI-R and our own statistical model contained 62 and 66%, respectively. For further details, see table S6.

## DISCUSSION

Risk assessment is “the engine that drives” a federal prison reform bill recently signed into law (*19*) and a component of many jurisdictions’ efforts to reduce incarceration rates without compromising public safety (*20*). When risk is a legally relevant factor, judges, correctional authorities, and other professionals have been advised to consider RAIs when making decisions. The assumption is that RAIs predict reoffending better than unaided human judgment.

Dressel and Farid’s findings challenge this assumption in a setting where risk information is constrained, feedback on accuracy is provided across many trials, and base rates of recidivism are balanced. In the present series of experiments, we examined the robustness of that result by manipulating these three features. We replicated Dressel and Farid’s finding that people perform as well as algorithms under the conditions that they investigate. However, we also found that algorithms tended to outperform humans in settings where decision-makers have access to extensive information and do not receive immediate feedback and base rates are far from balanced, features of many real-world scenarios.

In general, our findings are consistent with much of the past research comparing human and algorithmic decisions (*21*–*23*); for crime-specific reviews, see (*24*, *25*). For example, on the basis of a meta-analysis of 41 studies, Ægisdóttir *et al.* (*21*) found that statistical methods were reliably superior to humans in predicting a range of outcomes. For predicting violence and other criminal behavior specifically, they note that algorithms were “clearly superior to the clinical [human] approach.” Similarly, several studies conducted with judges and correctional officers indicate that algorithms and RAIs outperform their professional judgment in predicting recidivism [cf. Goel *et al.* (*26*) for an overview].

Against this backdrop, Dressel and Farid’s finding was unexpected. Their work, however, helps provide hints about the conditions under which humans may perform as accurately as algorithmic RAIs. Although we could not examine every possibility in our experiments, our results point toward two sets of conditions that influence the relative accuracy of humans. First, when base rates are unbalanced, our results suggest that providing people with feedback can improve their classification accuracy to rival that of algorithms. We explicitly informed all participants in our experiments about the base rate of recidivism, but classification accuracy improved only among the subset of participants who also received trial-by-trial outcome feedback. Across trials, people who received feedback, compared to those who did not, became less likely to guess that an individual would reoffend.

In justice settings, this feedback is exceedingly rare. Judges may never find out what happens to individuals that they sentence or for whom they set bail. Theoretically, jurisdictions could address this gap and create a more kind learning environment by requiring that judges express and record their intuitive estimates of risk and by providing regular feedback on past predictions. With that information, judges could, for example, see the actual postrelease recidivism rate of those that they had deemed “high risk.” As in our experiments, this feedback could correct tendencies to overpredict recidivism. Improving judges’ ranking accuracy, however, could prove more difficult.

Second, our results suggest that people can predict recidivism as well as statistical models if only a few simple predictive factors are specified as inputs, as was the case in Dressel and Farid’s study. In this context of streamlined inputs, the accuracy of models and humans (without feedback) was largely interchangeable. In contrast, when inputs were enriched with additional predictive factors, models outperformed human judgment. This was not because the additional risk information compromised human judgment (people’s performance did not differ much in streamlined versus enriched conditions). Instead, it was because models made better use of the additional information than did humans.

We note, however, that even in the enriched condition, the additional information that we provided was still relevant for recidivism prediction, as it was included in the LSI-R risk assessment tool. Like Dressel and Farid’s study, then, our experiments compare the accuracy of algorithms and RAIs with that of structured human judgment, which has been found to consistently outperform unstructured judgment in predicting violence and other recidivism [cf. Goel *et al.* (*26*) for a summary]. To better represent human judgment in justice settings, we hope that future studies provide even more realistic and complete inputs for prediction, including irrelevant or potentially distracting information (*27*). Still, together with past work, our results support the claim that algorithmic risk assessments can often outperform human predictions of reoffending.

## MATERIALS AND METHODS

### Datasets

Our two COMPAS experiments were based on a single dataset that is composed of 7214 defendants from Broward County, FL (*4*) who were scored with COMPAS for both risk of recidivism and risk of violent recidivism. While the Dressel and Farid (*3*) study only considered human predictions of overall recidivism, we additionally considered predictions of violent recidivism. The COMPAS dataset contains individual-level demographic information (age and gender); criminal history (current charge and number of past arrests); and whether or not each defendant was arrested for a new crime or, separately, a new violent crime within 2 years of COMPAS scoring, excluding any initial detention period. We restrict to the same randomly selected subset of 1000 defendants used by Dressel and Farid.

We also made use of two LSI-R (*11*) datasets: one containing 311 individuals under correctional supervision, drawn from Lowenkamp and Latessa (*28*), and another containing 1954 individuals on probation, drawn from Flores *et al.* (*29*). These datasets include numerical scores for 10 risk factors or subscales of the LSI-R (e.g., criminal history, antisocial peers, and substance use). All individuals in these datasets were followed for a minimum of 1 year after the LSI-R was administered to assess recidivism, which was for any arrest in the correctional supervision dataset and any reincarceration (a low base rate phenomenon) in the probation dataset.

### Vignettes

For the COMPAS data, we generated short vignettes to show study participants following the method of Dressel and Farid. In particular, participants were given a brief description of each individual’s age, gender, current criminal charge, and number of past arrests. For the LSI-R data, we first created several one-sentence descriptions for each of the 10 LSI-R factors and risk levels, discretized to “low,” “medium,” and “high.” For example, one such sentence for low risk on the criminal history scale was “He has never been convicted of a prior offense.” These sentences were created by consulting the LSI-R scoring manuals for each jurisdiction. Participants in the streamlined condition were presented with the individual’s age, gender, and number of past arrests, as in the COMPAS experiments. (Current charge was not available in the LSI-R datasets.) Participants assigned to the enriched condition were shown a brief paragraph describing an individual’s age and gender, followed by one-sentence descriptions for each of the 10 LSI-R risk factors, displayed in random order.

### Participants

Study participants were recruited through Amazon’s Mechanical Turk. The study was advertised as follows: “You are invited to participate in a research study on predicting criminal behavior. You will be presented with a series of descriptions that requires a classification decision (e.g., assessing an individual’s risk of recidivism). We will not ask or record any personal information.” Each participant was asked to assess the recidivism risk for 50 individuals randomly selected from the corresponding dataset. As in Dressel and Farid (*3*), we additionally asked participants to answer two attention checks at different points in the experiment, and we only included responses from the 71% of participants who passed both attention checks in our analysis. Our four experiments were conducted in close succession over the course of several weeks, and participants were only allowed to complete one experiment.

Each participant received $1 for completing the study and a bonus of up to $5 based on performance. Following previous work (*30*), we measured performance via Brier scoring, an incentive-compatible payment scheme for eliciting probabilities (*31*–*33*). For each question *i*, the Brier score is 1 − (*Y_i_* − *p_i_*)^2^, where *p_i_* is the probability of recidivism reported by the participant, *Y_i_* = 1 if the individual described was indeed arrested for a new (violent) crime within 2 years of release, and *Y_i_* = 0 otherwise. A participant’s final score was computed by summing the Brier scores earned for the 50 substantive questions, excluding the two attention checks. Across participants and experiments, the average hourly compensation was approximately $25.

### Statistical models

For comparison to the existing tools (COMPAS and LSI-R), we fit logistic regression models on the same data that were made available to human participants. In the case of the COMPAS datasets, we used a separate training set of 6214 cases, which is the remainder after excluding the 1000 cases used as test data. For the LSI-R datasets, we used leave-one-out evaluation over all available data. Probabilistic predictions from the models were then rounded to the nearest value of the 30-point scale presented to participants in our experiment to ensure that the statistical models were evaluated under the same technical constraints.

### Computing AUC

To compute AUC for human predictions, we first calculated the AUC for each study participant and then averaged these results across all participants in a given dataset and treatment condition. We noted that Dressel and Farid collected only binary predictions, rather than probability estimates, from participants and, therefore, could not report AUC in this manner. Instead, for each vignette, they first computed the proportion of participants who predicted the individual would recidivate and then computed AUC across the set of vignettes on the basis of these proportions. Accordingly, the AUC values reported by Dressel and Farid represent the “wisdom of the crowd,” which often exceeds the average performance of individuals (*34*, *35*).

## Supplementary Material

http://advances.sciencemag.org/cgi/content/full/6/7/eaaz0652/DC1

Download PDF

The limits of human predictions of recidivism

## SUPPLEMENTARY MATERIALS

Supplementary material for this article is available at http://advances.sciencemag.org/cgi/content/full/6/7/eaaz0652/DC1 Fig. S1. Ranking performance of human predictions, statistical models, and existing tools. Fig. S2. A comparison between the classification accuracy of humans and existing tools. Fig. S3. Average classification accuracy over time with feedback. Fig. S4. Calibration plot for human responses. Table S1. Relative classification accuracy of humans without feedback. Table S2. Relative classification accuracy of humans with feedback. Table S3. Relative classification accuracy of humans with and without feedback. Table S4. Relative ranking accuracy of humans without feedback. Table S5. Relative performance of humans and models in the streamlined and enriched conditions. Table S6. Relative recall of humans without feedback.
