# Supplementary material for: The limits of human predictions of recidivism
Source: Sci Adv. 2020 Feb 14;6(7):eaaz0652. doi: 10.1126/sciadv.aaz0652 (PMC7021503; doi:10.1126/sciadv.aaz0652)
Supplement: http://advances.sciencemag.org/cgi/content/full/6/7/eaaz0652/DC1 [file supp_6_7_eaaz0652__index.html]

Science Advances | Science AdvancesAAASSearchScience AdvancesMenu

## Supplementary Materials

**This PDF file includes:**

- Fig. S1. Ranking performance of human predictions, statistical models, and existing tools.
- Fig. S2. A comparison between the classification accuracy of humans and existing tools.
- Fig. S3. Average classification accuracy over time with feedback.
- Fig. S4. Calibration plot for human responses.
- Table S1. Relative classification accuracy of humans without feedback.
- Table S2. Relative classification accuracy of humans with feedback.
- Table S3. Relative classification accuracy of humans with and without feedback.
- Table S4. Relative ranking accuracy of humans without feedback.
- Table S5. Relative performance of humans and models in the streamlined and enriched conditions.
- Table S6. Relative recall of humans without feedback.

Download PDF

**Files in this Data Supplement:**

- Adobe PDF - aaz0652\_SM.pdf
