# Supplementary material for: The limits of human predictions of recidivism
Source: Sci Adv. 2020 Feb 14;6(7):eaaz0652. doi: 10.1126/sciadv.aaz0652 (PMC7021503; doi:10.1126/sciadv.aaz0652)
Supplement: Download PDF [file aaz0652_SM.pdf]

Science Advances
AAAS

advances.sciencemag.org/cgi/content/full/6/7/eaaz0652/DC1

Supplementary Materials for

The limits of human predictions of recidivism

Zhiyuan "Jerry" Lin, Jongbin Jung, Sharad Goel*, Jennifer Skeem

*Corresponding author. Email: scgoel@stanford.edu

Published 14 February 2020, *Sci. Adv.* **6**, eaaz0652 (2020)
DOI: 10.1126/sciadv.aaz0652

This PDF file includes:

Fig. S1. Ranking performance of human predictions, statistical models, and existing tools.
Fig. S2. A comparison between the classification accuracy of humans and existing tools.
Fig. S3. Average classification accuracy over time with feedback.
Fig. S4. Calibration plot for human responses.
Table S1. Relative classification accuracy of humans without feedback.
Table S2. Relative classification accuracy of humans with feedback.
Table S3. Relative classification accuracy of humans with and without feedback.
Table S4. Relative ranking accuracy of humans without feedback.
Table S5. Relative performance of humans and models in the streamlined and enriched conditions.
Table S6. Relative recall of humans without feedback.

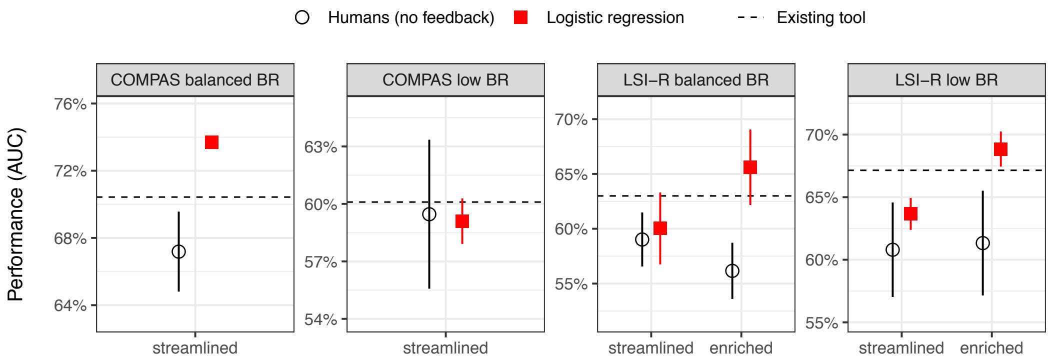

Fig. S1. Ranking performance of human predictions, statistical models, and existing tools. *Ranking performance, as measured by AUC, of: (1) human predictions without feedback; (2) logistic regression models that use the same information provided to study participants; and (3) the existing tools, COMPAS or LSI-R. Error bars indicate 95% confidence intervals.*

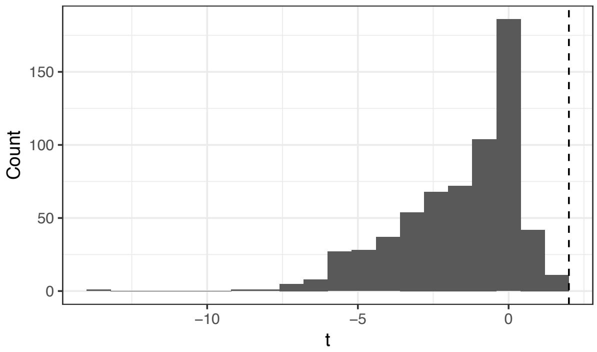

Fig. S2. A comparison between the classification accuracy of humans and existing tools. *Distribution of the t-statistic for the difference in classification accuracy between humans and existing tools (COMPAS or LSI-R) across all 645 participants. The vertical dashed line is at $t = 2$. None of the participants outperformed the existing tools by a statistically significant margin.*

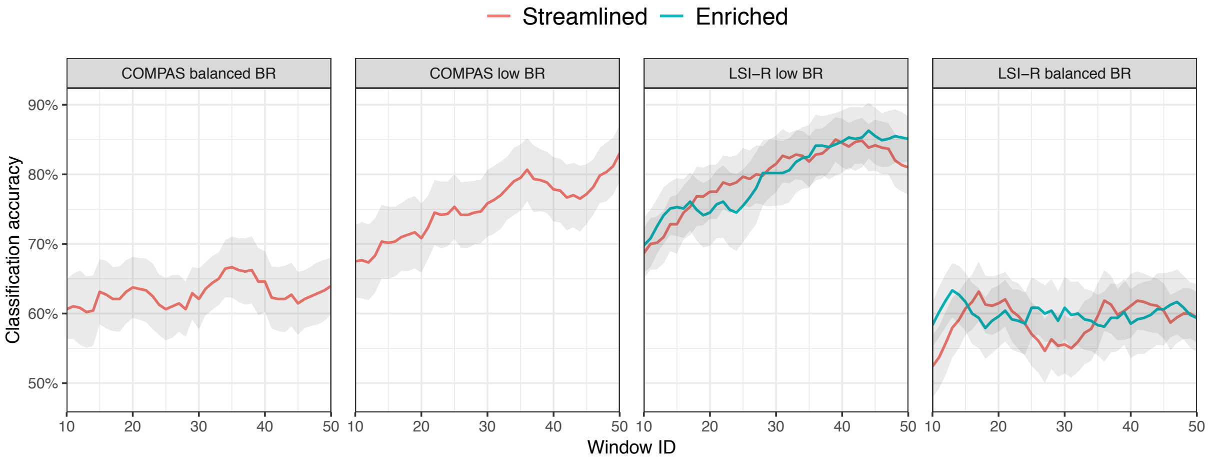

Fig. S3. Average classification accuracy over time with feedback. *Results are shown for a sliding window of 10 questions, where the window ID indicates the last question of that window. Humans recalibrated as a result of feedback, and we accordingly observed increasing accuracy. The largest improvements occurred for groups with low base rates. Grey bands indicate 95% confidence intervals.*

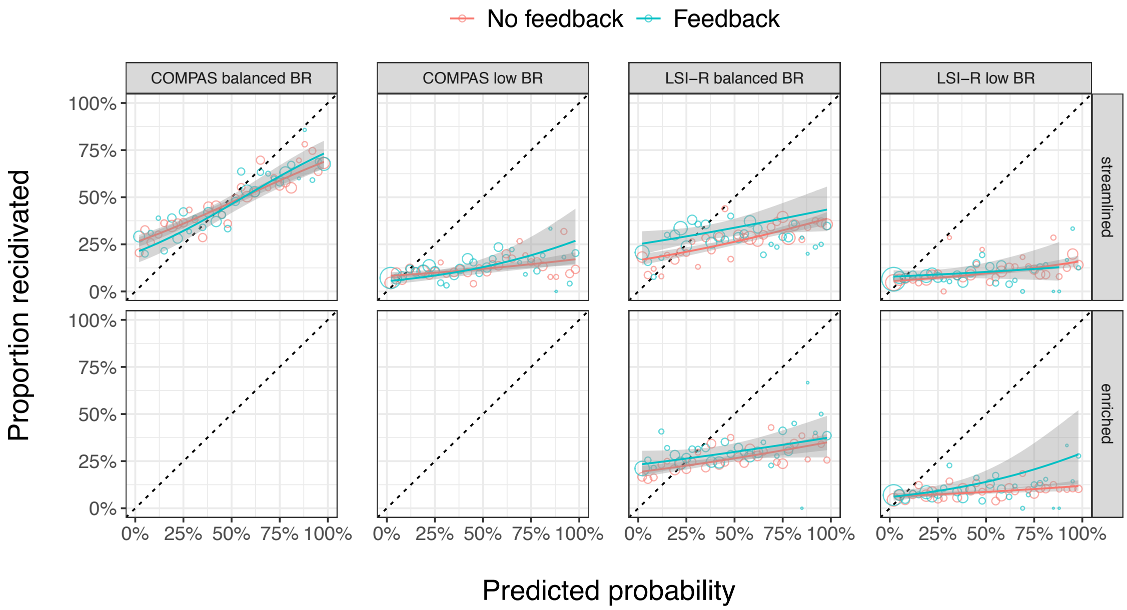

Fig. S4. Calibration plot for human responses. *Lines show a logistic regression of participants' estimated probabilities against the actual outcomes, and indicate that human predictions suffered from poor calibration. In the feedback conditions, we restricted to the final 10 responses to adjust for learning gains. In a re-analysis of Dressel and Farid's data, Bansak (36) likewise found evidence of poor calibration in human predictions of recidivism.*

Table S1. Relative classification accuracy of humans without feedback. *Difference in classification accuracy (in percentage points) between humans without feedback and: (1) existing tools (COMPAS or LSI-R); and (2) our own logistic regression models. In all cases, the algorithms outperformed the study participants by a statistically significant margin, with the standard error of estimates in parentheses.*

		Difference between existing tools and humans	Difference between our models and humans
Streamlined	COMPAS balanced BR	**0.03 (0.01)**	**0.06 (0.01)**
	COMPAS low BR	**0.30 (0.02)**	**0.30 (0.02)**
	LSI-R balanced BR	**0.24 (0.02)**	**0.24 (0.02)**
	LSI-R low BR	**0.34 (0.02)**	**0.34 (0.02)**
Enriched	LSI-R balanced BR	**0.17 (0.02)**	**0.16 (0.02)**
	LSI-R low BR	**0.32 (0.02)**	**0.32 (0.02)**

Table S2. Relative classification accuracy of humans with feedback. *Difference in classification accuracy (in percentage points) between humans with feedback and: (1) existing tools (COMPAS or LSI-R); and (2) our own logistic regression models. The algorithms outperformed humans in all cases, with bolded entries indicating statistically significant gaps and standard errors in parentheses.*

		Difference between existing tools and humans	Difference between our models and humans
Streamlined	COMPAS balanced BR	0.01 (0.02)	**0.04 (0.02)**
	COMPAS low BR	**0.06 (0.02)**	**0.06 (0.02)**
	LSI-R balanced BR	**0.12 (0.02)**	**0.12 (0.02)**
	LSI-R low BR	**0.10 (0.02)**	**0.10 (0.02)**
Enriched	LSI-R balanced BR	**0.12 (0.02)**	**0.11 (0.03)**
	LSI-R low BR	**0.06 (0.02)**	**0.06 (0.02)**

Table S3. Relative classification accuracy of humans with and without feedback. *Difference in classification accuracy (in percentage points) between participants who did and did not receive feedback. In all cases, feedback improved accuracy, with statistically significant differences indicated in bold.*

		Difference between feedback and no feedback
Streamlined	COMPAS balanced BR	0.02 (0.02)
	COMPAS low BR	**0.24 (0.03)**
	LSI-R balanced BR	**0.12 (0.02)**
	LSI-R low BR	**0.23 (0.03)**
Enriched	LSI-R balanced BR	0.05 (0.03)
	LSI-R low BR	**0.26 (0.03)**

Table S4. Relative ranking accuracy of humans without feedback. *Difference in ranking accuracy (AUC) between humans without feedback and: (1) existing tools (COMPAS or LSI-R); and (2) our own logisitic regression models. The algorithms outperformed humans in nearly every case, with bolded entries indicating statistically significant gaps and standard errors in parentheses.*

		Difference between existing tools and humans	Difference between our models and humans
Streamlined	COMPAS balanced BR	**0.03 (0.01)**	**0.06 (0.01)**
	COMPAS low BR	0.01 (0.02)	0.00 (0.02)
	LSI-R balanced BR	**0.04 (0.01)**	0.01 (0.02)
	LSI-R low BR	**0.06 (0.02)**	0.03 (0.02)
Enriched	LSI-R balanced BR	**0.07 (0.01)**	**0.09 (0.02)**
	LSI-R low BR	**0.06 (0.02)**	**0.08 (0.02)**

Table S5. Relative performance of humans and models in the streamlined and enriched conditions. *Difference in performance (as measured by classification accuracy and AUC) between the streamlined and the enriched conditions, for both humans without feedback and our logistic regression models. Positive values indicate better performance in the enriched condition, with standard errors in parentheses and statistically significant differences in bold. For ranking accuracy (AUC), our models improved by a statistically significant margin in both datasets when provided with enriched information, but the study participants did not.*

		Accuracy	AUC
Humans	LSI-R balanced BR	**0.07 (0.02)**	-0.03 (0.02)
	LSI-R low BR	0.02 (0.03)	0.01 (0.03)
Models	LSI-R balanced BR	-0.01 (0.02)	**0.06 (0.02)**
	LSI-R low BR	0.00 (0.00)	**0.05 (0.01)**

Table S6. Relative recall of humans without feedback. *Difference in recall-at-50% (in percentage points) between humans without feedback and: (1) existing tools (COMPAS or LSI-R); and (2) our own logistic regression models. Recall-at-50% is the proportion of recidivists in the dataset that are contained in a list of the 50% of individuals deemed riskiest by a particular method. The algorithms outperformed humans in all cases, with bolded entries indicating statistically significant gaps.*

		Difference between existing tools and humans	Difference between our models and humans
Streamlined	COMPAS balanced BR	**0.04 (0.01)**	**0.07 (0.01)**
	COMPAS low BR	0.02 (0.02)	0.02 (0.02)
	LSI-R balanced BR	**0.07 (0.01)**	0.04 (0.03)
	LSI-R low BR	0.03 (0.02)	0.03 (0.02)
Enriched	LSI-R balanced BR	**0.05 (0.01)**	**0.09 (0.03)**
	LSI-R low BR	**0.15 (0.02)**	**0.21 (0.03)**
